# Some considerations about acquired adult and pediatric cholesteatomas

**DOI:** 10.1016/S1808-8694(15)31212-X

**Published:** 2015-10-20

**Authors:** Cristina Dornelles, Sady S. da Costa, Luíse Meurer, Cláudia Schweiger

**Affiliations:** 1 Master in Medical Sciences, Pediatrics, Ph.D. studies under course, Program of Post-graduation in Medical Sciences, Pediatrics - UFRGS, Biologist, Centro de Otite Média do Brasil.; 2 Otologist, Ph.D. in Surgery, Joint Professor, Department of Ophthalmology and Otorhinolaryngology, Program of Post-graduation in Medical Sciences, Pediatrics and Post-graduation in Medical Sciences: Surgery, Federal University of Rio Grande do Sul.; 3 Pathologist, Ph.D. in Sciences, Gastroenterology, Hired physician, Center of Pathology, Hospital de Clínicas de Porto Alegre; Advisor, Professor, Program of Post-graduation in Gastroenterological Sciences.; 4 Resident physician, Hospital de Clínicas de Porto Alegre. Service of Otorhinolaryngology - HCPA Service of Pathology - Hospital de Clínicas de Porto Alegre - HCPA.

**Keywords:** cholesteatoma, perimatrix, collagen

## Abstract

Authors debate about cholesteatomas, from the first time this word was employed, by Muller, in 1838, until the recent updates. They dissert about its definition, etiology and pathology and present basic concepts about its biology. They also make a wide review about pediatric cholesteathoma, its epidemiology and biology, and compare it with adult cholesteatoma. Finally, they describe some articles about ossicle chain erosion and its correlation with cholesteatoma perimatrix, collagen and collagenase.

## The word

The word “cholesteatoma” was used for the first time by the German anatomist Johannes Mueller, in 1838^1^. The roots of this word mean *cole* - cholesterol; *esteado* - fat; *oma* - tumor, that is, a tumor in which we have fatty tissue and cholesterol crystals. Etymologically, this term is completely incorrect, and it is considered the second wrong term in otology (the first one is acoustic neuroma, given that it is in fact a vestibular nerve Schwannoma) ^2^. The use of this denomination is inappropriate because cholesteatoma is originated from the keratinized squamous epithelium of the tympanic membrane and/or external auditory canal, without presence of cholesterol crystals or fat in its structure, in addition to the fact that the tumor nature is completely discussable.

Other suggested denominations were pearl tumor, by Cruveilhier, in 1829; *margaritoma*, by Craigie, in 1891, epidermal *cholesteatoma by* Cushing, in 1922, *epidermoid by* Critchley and Ferguson, in 1928 and keratoma, by Shuknecht, in 1974; all these terms even though they are more appropriate and descriptive, are not employed and the term cholesteatoma is widely used by otologists.

## Definition

Cholesteatomas were defined by Friedmann^3^, in 1959, as cystic structures recovered with stratified squamous cell epithelium, laying over a fibrous stroma of variable thickness, which can contain some elements from the original mucous lining.

Schuknecht^4^, in 1974, defined them as accumulation of exfoliated keratin inside the middle ear or any pneumatized area of the temporal bone, originated from the keratinized squamous epithelium. Informally, the author stated that the cholesteatoma could be characterized as “skin on the wrong side”.

## Epidemiology

Annual incidence of cholesteatoma ranges around 3 in 100,000 in children and 9 in 100,00 in adults, and it is more predominant in male 5, 6.

Epidemiological data show high prevalence of cholesteatoma among Caucasian people, followed by African descendents, and it is rarely detected in Asian population. According to Ratnesar^7^, this prevalence is very low in Inuit Eskimos, suggesting that their anatomical morphological characteristics may facilitate the aeration of the middle ear and prevent sequels of chronic otitis.

In the Ambulatory of Chronic Otitis Media, Hospital de Clínicas de Porto Alegre (AOMC-HCPA), out of 450 patients followed up since August 2000, 30% had cholesteatomatous chronic otitis media (CCOM), presenting bilaterally in 12% of the total sample. Out of the patients with CCOM, 45% were aged up to 18 years, considered to be pediatric patients. As to gender, we found 70% of male cases ^8^.

## Classification

Cholesteatomas are normally classified as congenital and acquired and they are subdivided into primary and secondary.

Congenital cholesteatomas are epithelial remains found in ears with intact tympanic membrane and without previous history of infections ^9^. According to Valvassori^10^, they are found in four regions of the temporal bone: tympanic-mastoid, petrous apex, cerebello-pontine angle and jugular foramen. There is a fifth explanation, described by Sobol^11^, that reported the existence of small epithelial pearls between the tympanic membrane layers.

Acquired primary cholesteatomas are resultant from tympanic retractions that would accumulate the desquamated epithelium and would lose their self-cleaning power. Secondary cholesteatomas would be formed from the migration of the epithelium through a marginal perforation in the tympanic membrane (Costa et al., 1999).

Meyerhoff and Truelson^6^ tried to classify cholesteatomas according to the pathophysiology, location, ossicle defects and presence of complications, dividing them into congenital and acquired, and the latter into primary, secondary and tertiary.

Tos^6^ proposed another classification based on site of origin of cholesteatoma, which considers it as an important factor for the surgical procedure and the prognosis. This taxonomy presents three categories:

1. Attic Cholesteatoma - retraction of the flaccid pars or Shrapnell membrane, extending to the attic, going through the aditus and eventually reaching the antrum, mastoid or tympanic cavity.
2. Tympanic sinus Cholesteatoma - posterior-superior retraction or perforation of pars tense, extending to the tympanic sinus and posterior portion of the tympanic membrane.
3. Pars tense Cholesteatoma - retraction and total adhesion of the pars tense of tympanic membrane involving the tympanic orifice of the auditory tube.

Saleh and Mills^13^ proposed another classification, according to the site affected by the cholesteatoma, characterized as follows:

S1 - if the cholesteatoma is restricted to the site where it had started;
S2 - when the disease extends to other site;
S3 - if it affects three sites;
S4 - if it is installed in four sites;
S5 - to cases in which the primary site is affected plus four or more are also involved.

The same authors distinguished seven sites used to this classification: attic and antrum, middle ear, mastoid, auditory tube, labyrinth and middle fossa. The staging is a practical solution to describe disease extension and it is clinically relevant, which may be applied to all lesions except to the petrous apex, which cannot be diagnosed by otoscopy.

Saleh and Mills^13^ also presented a classification of the ossicle chain condition, based on the descriptions by Wullstein (1956) and Austin (1969), through the following score:

0 - if the ossicle chain is intact;
1 - if incus is eroded and without chain discontinuity;
2 - if incus and stapes suprastructures are eroded;
3 - if the malleus head and incus are absent and stapes superstructure if eroded.

As to preoperative complications, Saleh and Mills^13^ classified cholesteatomatous chronic otitis media as:

C0 - when there are complications;
C1 - to the occurrence of complications;
C2 - to the existence of two or more.

As to complications, the authors consider lateral semicircular canal fistula, facial palsy, total sensorineural auditory loss, sinus thrombosis and intracranial invasion.

Deep understanding about the pathogenesis of the middle ear cholesteatoma is particularly important, given that the destructive nature is responsible for many of the referred complications. The trend to bone erosion of cholesteatomas and the lack of non-surgical treatment justify the importance of the investigation of basic mechanisms related to development of cholesteatomatous chronic otitis media.

## Etiopathogenesis

Ferlito^14^ described that three disposing conditions would be necessary for the development of cholesteatoma: a) the junction of two different epithelia in the auditory opening; b) the chronic destruction of the middle ear submucous layer by infectious and inflammatory processes; c) the scaring process or the proliferation phase.

**Chart 1:**
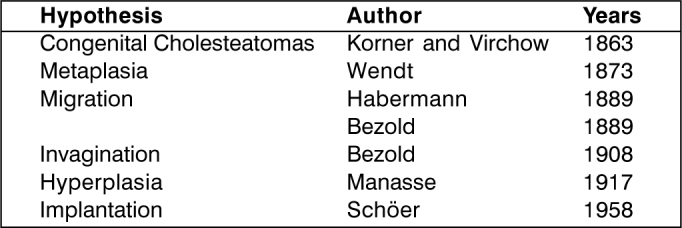
Main hypotheses for the etiopathogenesis of cholesteatomas.

However, the etiopathogenesis of cholesteatomas is still very much discussed and basically there are six main hypotheses (Chart 1), which have generated controversy for over 100 years.

There are a number of studies about the pathogenesis of cholesteatomas, but there is still a lot to be clarified ^15^. As observed in AOMC-HCPA and in many different studies of this group 8, 16, 17, 18, 19, 20, 21, 22 these hypotheses would not individually explain the pathogeneses of all cholesteatomas.

The existence of congenital cholesteatomas and the onset of cholesteatoma by invagination and implantation is unequivocal, but these situations would not be responsible for all cases of CCOM. We believe that the pathogenesis of cholesteatoma in fact would involve many different aggregated hypotheses (Chart 1), and there may be an overlapping of two or more of them in each patient.

As a consequence of this fact, it seems that the hypothesis of the continuum, advocated by Michael Paparella^23^, in 1970, is close to a multifactorial and more comprehensive conception of pathogenesis. According to it, otitis media would exist throughout a continuous series of epithelial and subepithelial events, in which after the initial insult, the serous or purulent otitis would become serummucoid, mucoid or finally, if there were no spontaneous or therapeutic regression of the condition, there would be chronic transformation. We can say, based on this hypothesis, that the cholesteatoma is a complex of one single middle ear pathology, chronic otitis media, and not an isolated event.

## Structure

Macroscopically, cholesteatoma is a round or oval cystic lesion with variable configuration and size. Ferlito et al. ^14^ characterized cholesteatoma as an epidermoid cyst of independent and progressive growth with destruction of adjacent tissues, especially the bone tissue, with tendency to recurrence.

The advent of transmission electron microscopy allowed many advances in the knowledge about cell structure. Using this instrument, Lim and Saunders^24^ in 1972 presented a detailed histological description of cholesteatomas. They described that the cholesteatoma has a keratinized stratified squamous epithelium with four layers identical to normal epidermis (basal, spinosum, granulosum and corneal), Langerhans cells (in larger amounts than in normal epidermis) and keratin-hyaline granules. They named this epithelium as cholesteatoma matrix. They also observed the presence of connective tissue, containing collagen fibers, fibrocytes and inflammatory cells, which was named perimatrix, which is in most of the cases in contact with squamous or ciliated cylindrical cells, remains from the original middle ear mucosa. In some cases, despite the fact that the perimatrix is absent at optical microscopy, it was present when studied under transmission electron microscopy, proving to be extremely thin, with collagen fibers that were practically absent and containing sodium carbonate crystals. In the study conducted by Paludetti et al. ^25^, the perimatrix consisted of granulation tissue or inflamed subepithelial connective tissue.

According to Milewski et al. ^26^, the growth of cholesteatoma could require angiogenesis in the perimatrix connective tissue, and cells and substances of the scaring cascade would have an important role in the development and growth of cholesteatoma. These processes would involve the fibroblastic growth factor b (b-FGF), which according to some authors, could stimulate collagenase. They also suggested that persistence of inflammation would cause a permanent process of scaring in the perimatrix, proliferation of fibroblasts (granulation tissue) and epithelium (matrix).

Ferlito et al. ^14^ described the perimatrix as the most peripheral portion of the cholesteatoma, comprising granulation tissue or inflammatory subepithelial connective tissue, with lymphocytes, hystiocytes and neutrophils. Sprekelsen et al. ^27^ stated that the matrix and perimatrix, in normal or pathological tissues, are formed by type IV collagen, tenascin, fibronectin, b-FGF and metalloproteinase (MMP). According to Jacob et al. ^28^, the increment in proliferation of the cholesteatoma matrix would be the result of the inflammation process, suggesting that the perimatrix would be the main factor of cholesteatoma development.

**Figure 1:**
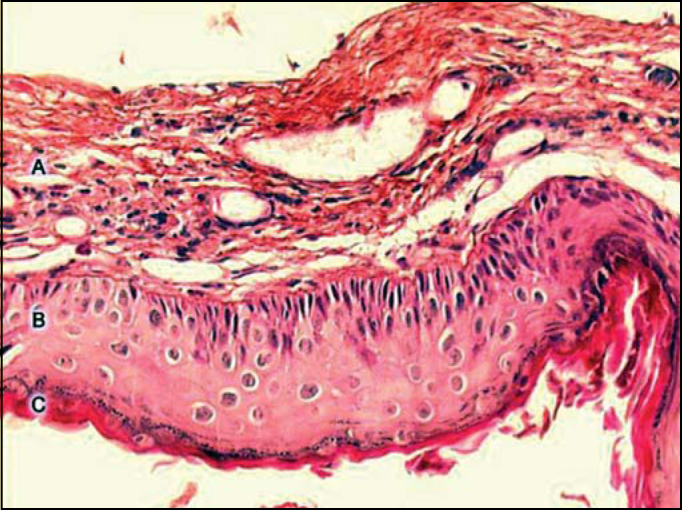
Digitalized image of the lamina, cross section of a cholesteatoma, stained with Hematoxylin-Eosin. We can see three forming parts: A - perimatrix, B - matrix, C - cystic content.

In summary, we can define the perimatrix as an inflammatory network that involves the cholesteatoma. Figure 1 presents a histological section of a cholesteatoma, in which we can see the structural components: perimatrix, matrix and cystic content.

Pereira et al. ^29^ studying 31 cholesteatomas - 20 adults and 11 children, found nine specimens without perimatrix, visible under optical microscopy, two in adults and seven in pediatric cases. These findings corroborate those reported by Lim and Saunders^24^, which confirmed that many perimatrixes can only be seen under electron microscopy. These authors suggest that a possible cause of this reduction in collagen fibers could be the result of a differential action of collagenase.

Hamsei et al. ^30^ analyzed 21 cholesteatomas through polymerase chain reaction (PCR), immunohistochemistry and histology, with the objective of investigating stimulation factors and differentiation of osteoclasts into cholesteatoma, using skin from the external acoustic canal as control. The immunohistochemical analysis demonstrated an increase in osteoclast and macrophage precursor cells in cholesteatomas. The perimatrix analysis showed that in these regions of the cholesteatoma, there are all factors necessary for osteoclastogenesis and for stimulation of bone reabsorption.

## Biology of Cholesteatoma

The study of cells under optic and electron microscopy may give us the mistaken impression that they are static structures. However, to the contrary, many processes and movements are constantly happening inside the cells, occurring in some tissues quicker than in others. It is easy to understand that as cells are getting differentiated, and parallel they acquire some structural and physiological particularities.

Epithelia are tissues with limited life, with constant renewal, resulting from continuous mitotic activity. The speed of cell replacement is variable, and it may range from 2 to 50 days, depending on the tissue to be considered ^31^.

The connective tissue, in turn, comprising cholesteatoma perimatrix, presents a growth process that is more complex because it is formed by different types of cells - fibroblasts, macrophages, mast cells, plasmocytes, leukocytes - separated by abundant intercellular material. Richness of this material is one of its most important characteristics. It is a part with defined microscopic structure - connective fibers - and by another non-structured portion - a substance that is fundamentally amorphous ^31^. Owing to this complexity, one could expect that the renewal process of this tissue would be quite complex.

Cholesteatomatous chronic otitis media could be the result of an uncontrolled cell proliferation; more simply, in this pathology there is growth of keratinized squamous epithelium cyst, the cholesteatoma^14^. It could be considered as a disorder of the control of cell growth, comprising a series of complex and dynamic events involving cell and extracellular components with affections to their biological behavior, such as keratinocyte deregulation ^32^, which present a hyperproliferative growth and cell differentiation affections. However, it is not known for sure whether this lack of control is caused by defect to the genes that control the proliferation, by cytokines released by inflammatory cells or by mechanisms still unknown ^12^. Thus, determining the existence of defects in its biology, biochemistry and genetics is essential to learn about the pathogenesis.

As previously described, the capacity to invade, migrate, modify the differentiation, proliferation and recurrence of cholesteatomas is quite similar to neoplasms, but there is reluctance between researchers about accepting the inclusion of cholesteatoma in this category 33, 34.

For cholesteatoma to be considered a neoplastic lesion it is necessary to have evidence of genetic instability; it may be manifested through DNA affection or specific chromosomic abnormalities. In 1995, Shinoda and Huang^35^ detected the protein p53 in cholesteatomas, suggesting that they could be tumors. However, Desloge et al. ^33^ demonstrated that there were no DNA affections, ruling out this hypothesis.

Given that the reported studies have not detected any genetic instability in these lesions, we should investigate another possible reason for the development of CCOM, being necessary to ask about the origin of keratinized squamous epithelium in cholesteatomas. To study this issue, many investigations using immunohistochemical analysis have been performed to compare the location of differentiation markers in cholesteatomas and on the skin of the external auditory canal. Owing to the properties of cytokeratins, they have been considered by many investigators 36, 37, 32 as the best instruments to this end.

Cytokeratins are proteins that form one of the two categories of intermediate filaments, located on the cytoplasm of epithelial cells; they have 20 subclasses, and their expression is dependent on the type of epithelium and stage of differentiation^38^. Pereira^39^, Albino et al.^40^ and Kim and Chung ^41^ reported that matrix of cholesteatomas expresses cytokeratin 16 (CK16) on suprabasal layers, being that the expression of this protein filament is characterized by hyperproliferative epithelium. Lepercque et al. ^42^ described that CK16 does not show on the normal epithelium unless it is in areas under pressure and friction, or on the recover epithelium of hair follicles. According to Broekaert et al. ^43^, CK16 is expressed in specific regions, such as tympanic ring and medial and inferior regions of the external acoustic canal. Pereira et al. ^29^ stated that the presence of CK16 in the matrix of the cholesteatoma could indicate a hyperproliferative behavior, similar to hyperproliferative epidermal disease. According to Albino et al. ^40^, cholesteatoma is formed as a result of the attempt to repair the lesion, which could explain the presence of CK16, characterizing this epithelium as immature with predominance of cell proliferation. Kujipers et al. ^37^ analyzed the pattern of cytokeratins and suggested that the cholesteatoma matrix is not a result of a metaplastic change. In the study, they found an epithelium similar to that of the tympanic membrane and to the skin of the external auditory canal, but in different stages of proliferation, depending on the level of inflammation present.

A characteristic signal of cholesteatoma is the infiltration in the perimatrix of immune system cells. Piltcher^44^, in his thesis about cytokines in chronic otitis media with effusion, stated that in addition to the known risk factors, such as auditory tube dysfunction and infections, many research studies about otitis media have been directed to the study of different components of the inflammatory response. The essential issue is whether the inflammation should be considered only a process of defense or whether it has a role in perpetuation of cholesteatomatous chronic otitis media. Milewski^45^ suggested that inflammatory cytokines, fibroblasts and macrophages would be responsible for the origin, growth and bone destruction of cholesteatoma. Many cytokines and growth factors could be involved in the proliferation mechanism and development of cholesteatoma epithelium 46, 47. Tomita^48^ stated that there are many hypotheses that growth factors and cytokines, present in cholesteatomas, induce the activation of genes, such as *c-myc*, causing the deregulation of cell proliferation. Sudhoff et al. ^49^ investigated the distribution and expression of tumor growth factor (TGF-alpha), epithelial growth factor (EGF-R) and oncogene *c-myc* in normal epithelial cells of middle ear and in cholesteatomas. These factors were found in the cholesteatoma matrix, but not in normal cells. In addition to autocrine regulation of epithelium, owing to production of epithelial growth factor (EGF), cholesteatoma hyperproliferation could depend on the interaction of the subepithelial tissue and the inflammatory changes that occurred in this pathology. Sudhoff et al. ^50^ investigated the expression and location of growth factors of angiogenesis in 22 cholesteatomas in comparison to normal epidermis of external auditory canal (8) and to mucosa of normal middle ear (5), to identify some growth factors involved in the pathogenesis of cholesteatomas. These researchers found 5.3±1.2 vessels/mm^2^ on the normal skin and mucosa, whereas in the group with cholesteatoma there was 21.1±11.7 vessels/mm^2^, and the mean ranged according to degree of inflammation of perimatrix: 9.0±3.5 vessels/mm^2^ in grade I, 19.2±3.6 vessels/mm^2^ in grade II and 31.7±9.4 vessels/mm^2^ in grade III. They also reported a reduction in collagen type IV and laminin on the basal membrane of the cholesteatoma, comparing to the controls.

A common characteristic in the pathogenesis of many types of cholesteatoma is the presence of bacteria, consequently, a large amount of cytokines released by inflammatory cells resulting from the immune response. The presence of bacteria to promote a critical link between cholesteatoma and the host, preventing the neoformed epithelium to conclude its process of differentiation, which would promote a quiescent status, minimally proliferative, without migration or invasion in this step ^51^. The interactions between inflammatory cells and epithelium of the cholesteatoma could be responsible for the induction of aberrant biological characteristics of the pathology.

Chole and Faddis^51^ studied, under electron transmission microscopy, 24 human cholesteatomas and 22 cholesteatomas in Mongolian squirrels. In the human sample, 16 presented 541 histological findings consistent with biofilm bacteria, whereas the material from the animal study showed 21 evidences of bacteria. These findings could be related with the activity of cholesteatoma, especially in persistent or recurrent infections and with resistance of topical or systemic antimicrobials. The authors suggested that the cholesteatoma matrix is an ideal medium for the development of a mixed microbiological biofilm. The authors stated that even though the bacteria in the biofilm are resistant to antibiotics by different mechanisms used by plankton bacterial, the exact mechanism of resistance to bacteria colony in biofilm is still unknown.

Studies published so far presented many data about the biology of cholesteatomas, but many questions still remain. As previously referred, cholesteatomas present neoplastic characteristics (invasion, migration, change in differentiation), but to present, no indication of genetic instability has been detected in the structure, a fact that rules out the possibility of considering it a neoplasm. Another property that seems to be constant in cholesteatomas is its hyperproliferative activity, which might be a possible explanation to its aggressive characteristics and uncontrolled growth. In addition, stimuli of immune response, represented by cytokines related to perimatrix inflammatory cells, represent a strong candidate to the role of key player in this intricate plot of mechanisms. All these hypotheses make us consider the complexity involved in the biology of cholesteatomas and consequently the multitude of events related with its pathogenesis.

## Power of Bone Erosion

Cholesteatomas have great power of bone erosion 52, 53. They normally reach the ossicle chain and less frequently the head bones, including the most rigid bone of the human body, the labyrinth, which demonstrates its strong destructive action over the bone tissue. Partial or total destruction of ossicle chain is observed in about 80% of the patients with cholesteatoma, whereas in non-cholesteatomatous chronic OM there is erosion of ossicle chain in approximately 20% of the cases 54, 55. The mechanisms that lead to this increase in bone degradation and invasion are currently being investigated ^16^.

According to Fisch^56^, complications caused by cholesteatomas may be divided into two groups: intracranial ones - meningitis, abscesses and venous sinus thrombosis, and temporal bone ones - mastoiditis, labyrinthic fistula, facial nerve palsy, labyrinthitis and ossicle destruction.

Sadé and Berco^57^ performed a histological exam in 80 ossicles obtained in surgery and 41 of them were from patients with NCCOM and 39 from CCOM. Bone erosion was found in 42.5% of the ossicles of patients without cholesteatoma and in 84% of the patients with cholesteatoma, and the difference was statistically significant (P<0.001). In the study by Prescott^58^, 81 children with CCOM were followed up; out of a total of 96 cholesteatomas (15 bilateral), there were only 19 integral ossicle chains, 23 presented erosion of malleus and intact suprastructure, 51 had erosion of malleus and loss of suprastructure, and three were not described.

Sadé and Fuchs^59^ compared the findings of ossicle erosion in adults (age ≥ 14 years) to those found in children (age ≤ 13 years). The percentage of destruction of stapes and malleus were similar in both groups; incus presented significantly greater destruction in adults. Facial nerve palsy and labyrinthic fistula were equally more destroyed in adults. Dornelles et al. ^16^ performed a study of the description of middle ear findings in the transoperative period of 55 patients with chronic otitis media, followed in the AOMC-HCPA. Out of these patients, 49% had diagnosis of CCOM. In the sample, there were 66% of cases with involvement of ossicle chain: in cases of CCOM the rate was 96% whereas in NCCOM it dropped to 37%. The presence of cholesteatoma was associated with existence of two or more affected ossicles, as well as higher prevalence of absence or erosion of ossicles. These findings indicate that most patients with COM, submitted to surgical intervention, have ossicle chain involvement and that frequency and extension of impairment were much more related with presence of cholesteatoma. In our group, we categorized ages as: children up to 18 years and adults as of 19 years. When we analyzed the ossicle chain, observed in the transoperative analysis of patients with CCOM, we found 100% of impairment in children and 92% in adults. When we compared the ossicles separately, we reached 30% of affections in the malleus, 30% in the stapes and 90% in the incus, without statistically significant difference between adults and children. Upon repeating the analysis following the same age classification used by Sadé and Fuchs^59^, the results were different from the ones reached by the authors, because we did not find statistically significant differences when comparing ossicles in children and adults ^16^.

According to Swartz^60^, ossicle destruction is more common among complications of cholesteatomas and the type of destruction depends on origin and mode of expansion. According to the data, the ossicle chain is intact in only 26% of the attic cholesteatomas, and the long process of incus is the most affected region, followed by incus body and malleus head. In turn, pars tense cholesteatomas have power of erosion of 90%.

Bone absorption is stimulated by a variety of factors, including inflammation, local pressure, specific cytokeratins and keratin ^6^. The enzymatic concept in which enzymes of epithelial origin are considered responsible for bone destruction was defined by Abramson^61^, ^62^, ^63^, ^64^, which demonstrated the presence of collagenase and hydrolase in cholesteatoma, a hypothesis that was later confirmed by Thompsen^65^. Ferlito et al. ^14^ suggested that the destructive property of cholesteatomas - the bone erosion is caused by the production of collagenase by the components of squamous and fibrous epithelial tissues. It is not properly demonstrated yet whether mineralized bone can be absorbed by collagenase. Other agents were incorporated, such as tumor necrosis factor (TNF), interleukins (IL-1^a^) and prostaglandins (PGE_2_) 66, 67, 68, to the hypothesis of bone absorption by biochemical action, exclusively played by collagenolytic enzymes.

Peek et al. ^69^ studied the concentration of lipopolysaccharides, components of the membrane of gram-negative bacteria ^70^ in cholesteatomas and compared to the level found in samples of patients with NCCOM. They found higher concentrations in patients with cholesteatoma and suggested that these results would be related with high levels of bone reabsorption.

## Pediatric Cholesteatoma

There are some references supporting that cholesteatomas are more aggressive and would have less favorable prognosis in children than in adults 71, 72, 73, 74, 75. Smythe et al. ^76^ consider that clinical behavior of pediatric cholesteatoma is so different in adults that it should be considered a different disease.

Quaranta et al. ^77^ tried to check whether the clinical behavior of cholesteatomas in children would depend on histomorphological characteristics of the perimatrix. They compared the number of plasmocytes, lymphocytes, macrophages, granulocytes and giant cell in the perimatrix of samples taken from 30 patients aged less than 16 years with those of 30 adults, used as controls. The results suggested that in children the number of inflammatory mononuclear elements of the perimatrix would be higher than in adults, with evident activity of the collagenase enzyme. Based on this behavior, the authors suggested that the perimatrix characteristics could place an important role in the pathogenesis of cholesteatoma, and it could be one of the factors that justified the clinical differences between children and adults.

Bujia et al. ^73^ analyzed the expression of MIB1 (monoclonal antibody marker of cell proliferation) in 20 cholesteatoma in children, whose controls were 15 cholesteatomas in adults and skin of the external auditory canal. Immunohistochemical analysis showed normal indexes of epidermis in the canal and increased levels in both categories of cholesteatomas. However, the number of cells in proliferation was significantly higher in the pediatric group (p<0.01). When they compared infected cholesteatomas with non-infected ones, we did not find any significant difference, suggesting that the index of proliferation could be independent from external factors.

Conversely, Edelstein^78^ stated that pediatric cholesteatoma would be less expansive, which would take to smaller incidence of complications and that adults would be more susceptible to facial palsy, intracranial infection, labyrinthic fistula and ossicle erosion. Disagreeing with this author, Darrouzet et al. ^79^ suggested that fewer complications in the pediatric group would be caused by the fact that in this group the duration of the disease is on average below what is found in adults.

However, the consensus among most of the authors is that recurrent cholesteatoma, that is, development of a new cholesteatoma after surgical treatment, and residual cholesteatoma, which is originated from the growth of non-removed parts of the cholesteatoma during the surgery ^80^, are more common in the pediatric group and the average recurrence rate is 30%, compared to 3% to 15% in adults 78, 81. Prescott^58^ followed 81 children with CCOM in a cohort of 10 years and found a percentage of cholesteatoma recurrence of 12%. Lino et al. ^82^ conducted a study in 83 children with CCOM and found 25% of recurrence, whereas residual cholesteatoma was diagnosed in 42%. These authors suggested that recurrence of cholesteatoma could be intimately related with auditory tube dysfunction and that high rate of proliferation of matrix could be responsible for residual cholesteatomas.

Ruah et al. ^72^ suggested that persistence of mesenchymal and major inflammatory reaction observed in the flaccid pars and in the posterior-superior quadrant of pars tense of tympanic membrane, as well as collagen and elastic affections observed in purulent and serous otitis media, can represent a pathological justification for the typical development of cholesteatoma in children.

## Management of Cholesteatoma

Treatment of CCOM is essentially surgical. The primary objective is complete eradication of the disease, providing to the patient a dry and complication-free ear. The secondary objective, although not less important, is to preserve or improve the tympano-ossicle system function, whenever possible ^83^.

The first one is reached through meticulous removal of the whole cholesteatoma (including the matrix and perimatrix in the wall up technique) and other compromised tissues. To that end, a variety of surgical techniques has been used, but they are summed up into two basic ones, depending on the removal or sparing of the posterior wall of the external auditory canal: wall up or wall down mastoidectomy. Regardless of the technique, the secret to surgical success is complete eradication of the disease. The selection of the procedures is based on type, grade and extension of cholesteatoma; in the preoperative auditory assessment, including presence of associated complications, contralateral ear status, together with auditory tube function and level of mastoid pneumatization. This choice will also depend on general patients’ conditions, age, origin and profession ^1^.

**Chart 2:**
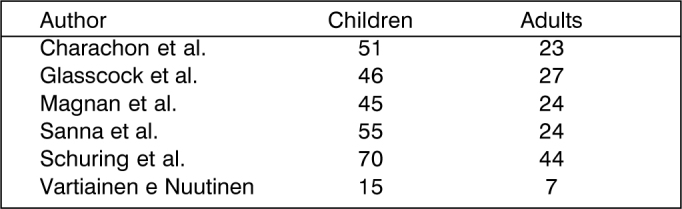
Incidence of residual and recurrent cholesteatomas, stratified by age group, as presented in the literature.

**Chart 3:**
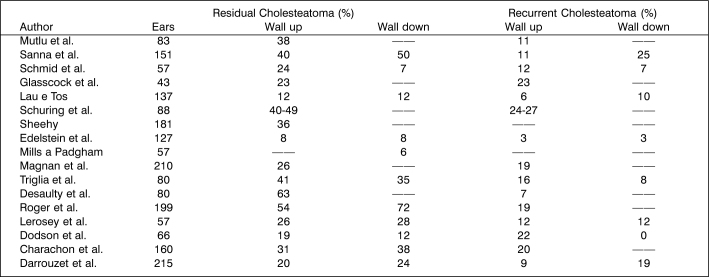
Frequency of residual and recurrent cholesteatomas, stratified by surgical technique as presented in the literature.

The wall down technique can be safer concerning eradication and prevention of recurrence, but it does not allow preservation of the anatomy and sometimes the level of preoperative hearing ^84^. We should bear in mind that the approach creates a cavity that will require careful and long medical follow up in addition to demanding general care for the whole life of the patient, characterizing a limiting factor for some sports such as swimming and scuba diving ^85^. However, the wall down technique, when compared to the wall up technique, presents less incidence of residual cholesteatoma ^86^, regardless of the fact that the rate varies a lot depending on the author 87, 89, 90, 91, 92. In all studies, it is always higher among children (Charts 2 and 3, below, adapted from Darrouzet et al. ^79^).

Since the first report of mastoidectomy technique, in 1649, by Riolanus, up to today, the surgery has been through a gradual evolution, supported by field studies and technological development. However, surgical management of cholesteatomas in children is still the key controversy among otologists 93, 94, 95. To define the best therapeutic strategy, especially in this special group of patients, it is necessary to consider many factors such as total eradication of the disease and preservation of auditory function; for the final decision, all factors should be properly considered and analyzed. The key issue is that up to present, no histological or biochemical differences have been found among cholesteatomas in different age ranges that could confirm or not the aggressiveness of CCOM in pediatric cases, a fact that would justify the use of less conservative surgical techniques.

## The next steps

One of the first steps in the advance of understanding and treating cholesteatomas is the interaction between clinical, histological and experimental studies.

The connection between these three lines of research will be essential to understand CCOM.
